# Maternal Adipocyte Connexin43 Gap Junctions Affect Breastmilk Lactose Levels and Neonate Growth in Mice

**DOI:** 10.3390/biology11071023

**Published:** 2022-07-07

**Authors:** Mingyang Huang, Anying Song, Xi Chen, Sarah Ishtiaq, Chunmei Wang, Darryl L. Hadsell, Qiong A. Wang, Yi Zhu

**Affiliations:** 1 Children’s Nutrition Research Center, Department of Pediatrics, Baylor College of Medicine, Houston, TX 77030, USA; mingyang.huang@bcm.edu (M.H.); xi.chen2@bcm.edu (X.C.); sarah.ishtiaq@bcm.edu (S.I.); chunmei.wang@bcm.edu (C.W.); dhadsell@bcm.edu (D.L.H.); 2 Department of Molecular Endocrinology, Diabetes and Metabolism Institute, City of Hope Medical Center, Duarte, CA 91010, USA; ansong@coh.org

**Keywords:** Connexin43, gap junction, obesity, lactation, breastmilk

## Abstract

**Simple Summary:**

Breastfeeding offers many health benefits for both mothers and infants. However, overnutrition and a steady increase in obesity in the U.S. has made it harder for many mothers to produce and express breastmilk. Moreover, the quality of breastmilk from obese mothers is frequently compromised in that it contains fewer nutrients and more inflammatory components. In this study, we used mice to model this phenomenon. We found that short-term high-fat feeding at the start of breeding reduces litter size and pups’ body weight. It also impairs adipocyte remodeling during lactation. Connexin43 is the primary building block for gap junctions in the adipose tissue. It is postulated to play an essential role in adipose tissue remodeling to accommodate mammary gland development and breastmilk production. Using genetically engineered mice without Connexin43 in their adipocytes, we demonstrated that the deletion of adipocyte Connexin43 affects the disappearance of adipocytes during lactation and affects milk composition, which is postulated to impair the pups’ growth. Altogether, this study suggests that increasing or enhancing adipocyte Connexin43 gap junctions may help obese mothers produce better breastmilk to support their neonates.

**Abstract:**

Breastfeeding offers a broad spectrum of health benefits for infants. However, overnutrition and a steady increase in maternal obesity in the U.S. have made it harder for many mothers to produce and express breastmilk, and the quality of milk from obese mothers is also frequently compromised. Adipocytes, the primary cell type in the non-lactating breast, display a drastic morphological and functional change during lactation in mice. Lipid-filled adipocytes undergo lipolysis, and lipid droplets disappear to provide fatty acids and energy for breastmilk production. Once the animal stops lactation, these lipid-depleted adipocytes return as lipid-laden cells. This dynamic remodeling of the tissue is likely the result of active intercellular communications. Connexin43 (Cx43) is the most abundant connexin in the mammary adipose tissue that makes up the gap junctions for direct intercellular communications. Its expression is increased during lactation and reduced in obese mammary adipose tissue, which is resistant to lactation-induced remodeling. However, whether Cx43 is required for adipocyte remodeling and breastmilk production to support neonates’ growth has not been established. In this study, we used doxycycline-inducible adipocyte-specific Cx43-deleted mice and demonstrated that adipocyte Cx43 played a vital role in determining the carbohydrate levels in breastmilk, which may subsequently affect neonates’ growth.

## 1. Introduction

In the most recent American Academy of Pediatrics (AAP) policy statement on “Breastfeeding and the Use of Human Milk,” the AAP reaffirms “its recommendation of exclusive breastfeeding for about 6 months, followed by continued breastfeeding as complementary foods are introduced, with the continuation of breastfeeding for a year or longer” [1]. The statement provides overwhelming evidence supporting the health benefits of breastfeeding for infants, including but not limited to reducing respiratory tract infections, gastrointestinal tract infections, necrotizing enterocolitis, sudden infant death syndrome and infant mortality, allergic disease, celiac disease, inflammatory bowel disease, obesity, diabetes, and childhood leukemia and lymphoma. Breastfeeding also supports and promotes the infant’s neurodevelopment [1].

Maternal obesity resulting from overnutrition has been steadily increasing in the U.S., posing an evolutionarily novel condition for mothers who want to breastfeed [2]. Several studies with different demographic cohorts demonstrated that mothers with high BMI have trouble lactating their young despite having the same breastfeeding intention [3,4,5,6]. Obese mothers produce a lower amount of breastmilk, and its quality is also compromised. For example, breastmilk from obese mothers usually has a pro-inflammatory fatty acid profile and decreased concentrations of unsaturated fatty acids and carotenoids, all of which have been shown to play critical roles in infants’ early visual and neurodevelopment [7]. To improve infant and maternal health, it is therefore crucial to better understand the biology of mammary development and lactation in overnutrition and obesity.

Besides the mammary gland structure, adipocytes contribute to most of the cells in a breast when evaluated using histological approaches, especially in its non-lactating state. However, the function and mechanisms through which adipocytes change their morphology during lactation remain poorly characterized. One hypothesis is that active intercellular communications via intercellular gap junctions might play a critical role. Gap junctions were the first mechanism that cells evolved to exchange cellular information and contents with neighboring cells [8]. In mammals, gap junctions are mostly made of connexin proteins [9]. Connexin43 (Cx43) is encoded by the gap junction protein alpha 1 (*Gja1*) gene and is the most studied connexin in the family [10]. Cx43 is also the most abundant connexin in the adipocytes [11,12,13], playing an essential role during adipogenesis [14] and in propagating signal inputs to the adipose tissue [12,15]. Its expression changes dynamically during adipocyte differentiation [14] and adipose depot development [16]. We first discovered that *Gja1* expression in adipocytes displays a dynamic pattern during the lactation process. Then, using a mouse model lacking *Gja1* in the adipocytes, we found that lactose concentration, but not total protein or triglyceride concentration, was significantly reduced in the breastmilk and that the growth of neonates feeding on this breastmilk was slowed. Together, these data suggest an important role for adipocyte Cx43 gap junction in regulating breastmilk macronutrients and neonates’ growth.

## 2. Materials and Methods

Mice. Cx43 (*Gja1*)-floxed mice (#008039) [17], TRE-Cre mice (#006234) [18], and APN-rtTA mice (#033448) [19] on the C57BL/6 background were purchased from the Jackson Laboratory. All animals were kept on a 12 h light–dark cycle in a temperature-controlled environment. Mice could freely access water and were fed either a standard chow diet, a 60% high-fat diet (HFD) (BioServ, S1850, Flemington, NJ, USA), or a 60% HFD containing 200 mg/kg doxycycline (BioServ, S6223, Flemington, NJ, USA).

Timed breeding, breastmilk extraction, and analysis. Age-matched control or adipocyte Cx43 knockout (APN-rtTA/TRE-Cre/*Gja1*^fl/fl^, abbreviated as Apn-Cx43-KO) females were mated at 12 weeks of age. Separate cohorts of C57BL/6J females were concurrently synchronized and mated to C57BL/6J males to produce age-matched cross-foster litters. On the day of parturition, each of the study dams were given weight-normalized litters of 6 one-day-old C57BL/6J wild-type pups. The endogenous pups were euthanized. On day 9 postpartum, the dams were separated from their litters for a period of 4 h to allow milk to accumulate, after which each dam was injected with 1 i.u. oxytocin (Sigma-Aldrich, O4375-250IU, St. Louis, MO, USA) [20]. The dams were then milked using an electric vacuum pump connected to a Pyrex bottle. Soft latex tubing for suckling milk was connected to a collection bottle that was connected to the Pyrex bottle with a two-hole rubber cap. The opening of the tube was placed on the mice’s teats, after which a vacuum was applied to obtain milk from each teat; this procedure was repeated for all 10 teats [21]. The breastmilk’s protein abundance was quantified using QPBCA reagents from Sigma-Aldrich (Cat. QPBCA-1KT, St. Louis, MO, USA). Lactose abundance was quantified using an assay kit (Sigma-Aldrich, Cat. MAK017, St. Louis, MO, USA) as a surrogate for the carbohydrate in the milk. Triglyceride content was quantified using Triglycerides Reagent (Fisher Scientific, Cat. TR22421, Waltham, MA, USA).

Adipocyte isolation. Collected inguinal adipose tissue was minced into small pieces and digested in 10 mL of adipose stromal vascular fraction cells isolation buffer and then placed in a shaking water bath for 1–1.5 h [12]. The digesture was filtered through 250 μm mesh, after which a room-temperature medium was added to a total volume of 20 mL. The mixture was centrifuged at 800× *g* for 1 min at room temperature and then the floating adipocytes (white cloudy layer) were collected.

Histology. The left and right inguinal mammary gland (both #5) were excised, fixed overnight in 10% PBS-buffered formalin, and stored in 50% ethanol. Tissues were sectioned (5 µm), rehydrated, and stained with hematoxylin and eosin (H&E) at the Pathology Core at BCM and COH. Microscopic images were taken on a ZEISS Axioscan scanner.

Western blotting. Protein extractions were performed as previously described [22], using primary antibodies Cx43 ((Santa Cruz Biotechnology, sc-271837, Dallas, TX, USA) (1:200 dilution) or (Santa Cruz Biotechnology, sc-6560-R, Dallas, TX, USA) (1:200 dilution)), β Actin (Cell Signaling, #3700) (1:2000 dilution), or α Tubulin (Santa Cruz Biotechnology, sc-53030, Dallas, TX, USA) (1:200 dilution). Protein abundance was detected using Goat anti-Mouse IRDye 680RD (Li-cor 926-68070, Lincoln, NE, USA), Goat anti-Rat DyLight 800 (Thermo Fisher SA5-10024, Waltham, MA, USA), or Goat anti-Rabbit Alexa 800 (Thermo Fisher A32735, Waltham, MA, USA) secondary antibodies at 1:10,000 dilutions. Antibody-decorated membranes were then visualized on a Li-Cor Odyssey infrared scanner (Li-Cor Bioscience, Lincoln, NE, USA), and the scanned data were analyzed using Odyssey Version 3.0 software (Li-Cor Bioscience, Lincoln, NE, USA).

qRT-PCR. RNA was isolated from frozen tissues by homogenization in Trizol Reagent (Invitrogen). A total of 1 µg of RNA was used to transcribe cDNA using a reverse transcription kit (Bio-rad, Hercules, CA, USA). qRT-PCR primers were obtained from Harvard PrimerBank [23] and are listed in Table 1. The messenger RNA levels were calculated using the comparative threshold cycle (Ct) method, normalized to gene *Rps16* or *Rps18*.

Immunofluorescence. Formalin-fixed, paraffin-embedded sections from either white adipose tissue or brown adipose tissue were blocked in PBST with 5% BSA. The primary antibody used was Cx43 (1:100 dilution; sc-6560-R, Santa Cruz); the secondary antibodies (1:250 dilution) used were Alexa Fluor 594 Donkey anti-Rabbit IgG (HCL). Slides were counterstained with DAPI. Fluorescent images were acquired using LSM510 (Zeiss) and were analyzed by ImageJ software.

Metabolic Measurements. Glucose tolerance tests were performed as described previously [22]: briefly, the mice fasted for 4–6 h, after which 10 µL/g of body weight glucose was injected. The final dose was 1.25 g/kg body weight. Blood glucose levels before and 15, 30, 60, and 120 min after glucose injection were measured using a glucometer. Intralipid challenge tests were performed as described previously [24]: Briefly, the mice fasted overnight, and then intralipid was orally gavaged at 15 µL/g body weight. Blood glucose levels before and 1.5, 3, and 6 h after intralipid administration were taken from the tail, and serum triglyceride levels were measured using Triglyceride reagents.

Statistical Analysis. Results are shown as mean ± SEM. For experiments with only two groups, the Student’s *t*-test was utilized. For studies with three or more groups, a one-way ANOVA was used, and for experiments with several groups with a balanced distribution of two factors, a two-way ANOVA test was used. Tukey’s test was used for post hoc analysis of comparisons within subgroups. *p* values < 0.05 were considered statistically significant.

## 3. Results

### 3.1. High-Fat Diet Feeding Impaired Adipocyte Remodeling and Difficulty in Supporting Neonate Growth during Lactation

**Figure 1:**
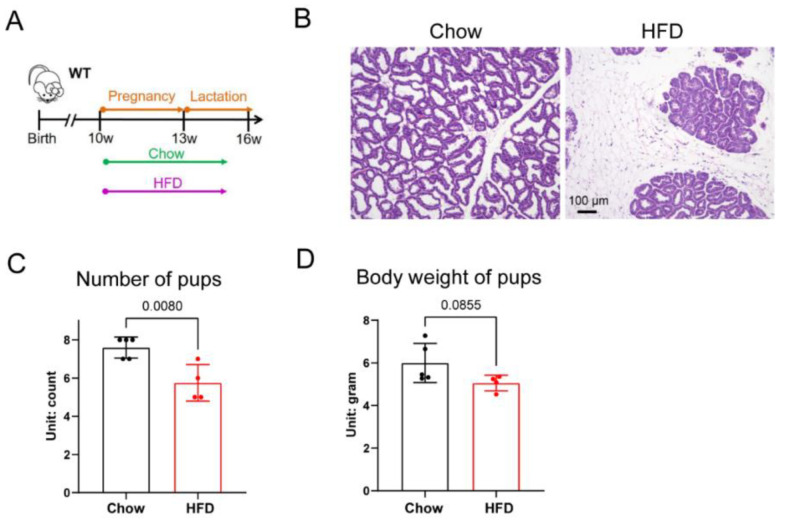
Obese female mice have impaired adipocyte remodeling and difficulty in supporting neonate growth. (**A**) Experimental treatment scheme to study how HFD feeding affects adipose tissue remodeling during lactation and the consequences for the pups. (**B**) H&E staining of mammary tissue from chow- or HFD-fed mice after lactating pups for 9 days. (**C**) Number of pups produced by dams under chow-fed or HFD conditions (*n* = 5 for chow group, *n* = 4 for HFD group). (**D**) Average body weight of pups nurtured by dams kept on chow or HFD (the average body weight of pups from one dam is regarded as one sample. *n* = 5 for chow group, *n* = 4 for HFD group). All data are mean ± SEM.

To model overnutrition during pregnancy and lactation, virgin female mice were fed a high-fat diet (HFD) during mating and lactation, starting at 10 weeks old (Figure 1A). Mammary adipose depot from dams fed a chow diet and lactating for 10 days showed a complete depletion of adipocyte lipid droplets (Figure 1B). In contrast, mammary adipose depot harvested from dams fed with a HFD diet contained patches of adipocytes (Figure 1B, HFD). The average number of pups per litter from the dams fed with HFD was also smaller (Figure 1C); yet, despite the reduced number of pups, their average body weight at 10 days old was also trending lower (Figure 1D). Together, these data suggest that impaired adipocyte remodeling during lactation in HFD-fed dams impaired lactation and support of their neonates’ growth.

### 3.2. Gja1 Is the Most Abundant Connexin Isoform in the Mammary Fat Pad and Obese Female Mice Have Reduced Adipose Tissue Gja1 Expression and Cx43 Protein Levels

**Figure 2:**
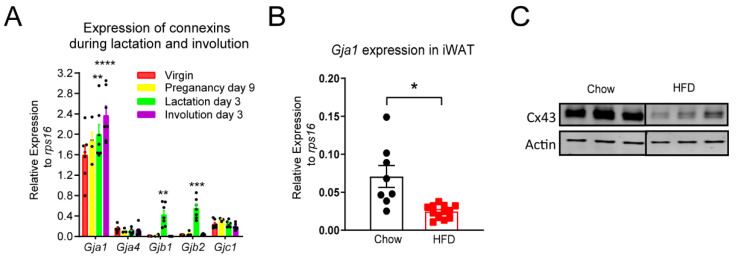
Mammary adipose tissue connexin expression at different stages of pregnancy/lactation and the effect of HFD feeding on mammary adipose tissue’s major connexin isoform *Gja1* expression. (**A**) Expression of *Gja1*, *Gja4*, *Gjb1*, *Gjb2*, and *Gjc1* in mammary fat pads from virgin mice, mice 9 days after successful mating (Pregnancy Day 9), mice 3 days post-parturition and lactating pups (Lactation Day 3), or mice 3 days post-weaning without a second round of pregnancy (Involution Day 3) (*n* = 7 mice). ** *p* < 0.01, *** *p* < 0.001, **** *p* < 0.0001, comparing to virgin group. (**B**) Expression of Gja1 in inguinal white adipose tissue (iWAT) from mice fed with HFD for 12 weeks was reduced. (*n* = 8 for chow, *n* = 12 for HFD). * *p* < 0.05. (**C**) Western blots showing a reduction in Cx43 protein abundance in inguinal mammary adipose tissue from mice fed with HFD for 12 weeks. All data are mean ± SEM. The original bolt can be found in Supplementary Figure S1.

We analyzed mammary tissue gene expression in each of the following: virgin mice, mice 9 days after successful mating (Pregnancy Day 9), mice 3 days post parturition and lactating pups (Lactation Day 3), and mice 3 days post weaning without a second round of pregnancy (Involution Day 3). This analysis showed that *Gja1* (encoding Cx43) is the most abundant gap junction gene transcript detected at all stages, and that its expression levels were increased on Lactation Day 3 and Involution Day 3 as compared to expression levels in virgin mice (Figure 2A). We also detected other connexin genes—*Gja4* (encoding Cx37), *Gjb1* (encoding Cx32), *Gjb2* (encoding Cx26), and *Gjc1* (encoding Cx45)—and their transcripts were all at least 10-fold lower than the *Gja1* in the mammary tissue. Interestingly, *Gjb1* and *Gjb2* expression levels were significantly increased on Lactation Day 3 and returned to basal levels on Involution Day 3 (Figure 2A). An analysis of mouse mammary tissue from mice kept on a HFD for 12 weeks showed a significant reduction in *Gja1* expression (Figure 2B) and Cx43 protein abundance (Figure 2C).

### 3.3. Adipocyte-Specific Cx43 Deletion Does Not Affect Glucose and Lipid Metabolism in Female Mice

**Figure 3:**
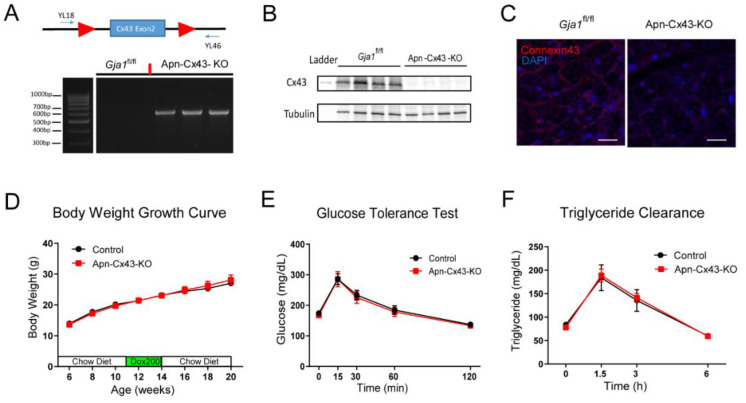
Generation of adipocyte-Cx43 knockout (Apn-Cx43-KO) mice and metabolic phenotyping. (**A**) Schematic of primers that only amplify recombined Gja1-floxed allele. YL18, CTTTGACTCTGATTACAGAGCTTAA; YL46, TATCATTCAAGCATCAATGATTATA. (**B**) Western blots showing complete deletion of Cx43 in adipocytes of Apn-Cx43-KO mice. (**C**) Representative immunostaining images showing deletion of Cx43 in inguinal white adipocytes from 4-week-old female mice. (**D**) Body weight development before and after Cx43 deletion induced by 3-week doxycycline diet (200 mg/kg) treatment in female mice (*n* = 8). (**E**). Glucose tolerance test of female control and Apn-Cx43-KO mice 6 weeks after doxycycline diet treatment (*n* = 8). (**F**) Intralipid challenge test of female control and Apn-Cx43-KO mice 7 weeks after doxycycline diet treatment (*n* = 8). All data are mean ± SEM. The original bolt can be found in Supplementary Figure S2.

To study the importance of adipocyte Cx43 gap junctions in adipocyte remodeling during lactation, we crossed APN-rtTA mice, TRE-Cre mice, and *Gja1*-floxed mice together to generate a compound transgenic animal: APN-rtTA/TRE-cre/*Gja1*^fl/fl^ (abbreviated as Apn-Cx43-KO). Littermate *Gja1*^fl/fl^ animals were used as controls. The adipocyte *Gja1* gene was deleted after adding doxycycline (200 mg/kg) into the diet, inducing CRE recombinase expression, which subsequently recombined two floxP sites [17]. PCR reactions using primers YL18 and YL46 amplified a fragment that could only be detected after CRE-mediated recombination, as non-recombined alleles would be too large to be amplified by the PCR condition (Figure 3A). Adipocytes were isolated from control and Apn-Cx43-KO mice. Cx43 was blotted and a complete knock out of the protein was observed (Figure 3B). The deletion of Cx43 in adipocytes was further confirmed by immunostaining of Cx43: As mammary tissue from control mice had intense Cx43 decoration in their cell membranes, the signal was largely absent from the tissue in Apn-Cx4-KO mice (Figure 3C). The deletion of Cx43 by switching the mice to a chow diet containing 200 mg/kg of doxycycline did not affect body weight development (Figure 3D). Deletion also had no effect on glucose tolerance (Figure 3E) or serum triglyceride levels after oral gavage of intralipid (Figure 3F), measured at 6 and 7 weeks, respectively, after the removal of the Dox200 chow diet. 

### 3.4. Adipose Tissue Cx43 Deletion Impairs Adipose Tissue Remodeling and Reduces Breastmilk Lactose Levels during Lactation

**Figure 4:**
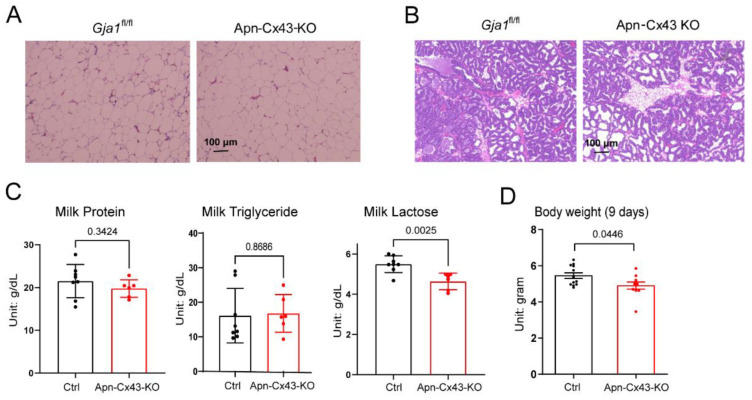
Adipocyte-specific Cx43 deletion reduces breastmilk lactose levels and impairs neonate growth. (**A**) H&E staining of mammary adipose depot from virgin mice. (**B**) H&E staining of mammary adipose depot from mice that have been lactating for 9 days. (**C**) Breastmilk macronutrient concentration (*n* = 8 for control (Ctrl) group, *n* = 6 for Apn-Cx43-KO group). (**D**) Average body weight of cross-fostered pups by control (Ctrl) or Apn-Cx43-KO dams (the average body weight of pups from one dam is regarded as one sample. *n* = 12 for Ctrl group, *n* = 10 for Apn-Cx43-KO group). All data are mean ± SEM.

Virgin Apn-Cx43-KO mice showed a similar morphology in their mammary adipose tissue (Figure 4A), but when the mice were lactating, their mammary adipose tissue showed patches of reminiscent adipocytes. This finding contrasts with the complete disappearance of adipocytes in control mice (Figure 4B). To understand whether the altered morphology of Apn-Cx43-KO mammary tissue affects breastmilk quality, we harvested breastmilk from control and Apn-Cx43-KO dams 9 days following the birth of their endogenous pups, after which the dams were immediately placed with 6 cross-fostered wild-type newborn pups. Our analysis of breastmilk macronutrients showed a similar level of protein and triglyceride concentration from that of the control and Apn-Cx43-KO dams (Figure. 4C). However, breastmilk lactose concentration was reduced by 15.6% (*p* = 0.0025) in the Apn-Cx43-KO group (Figure 4D). In parallel, the average body weight of cross-fostered pups 9 days after placement was reduced by 10.12% (*p* = 0.0446) (Figure 4D).

## 4. Discussion

Breastmilk is the best food and nutritional support for neonates in their early days of life, and improving milk quality is critical for supporting the health of the next generation [25]. However, the prevalence of maternal overnutrition and rising rates of obesity are making it harder for many women to express sufficient milk and maintain their milk’s nutritional quality [3,4,5,6]. During lactation, mammary adipocytes undergo drastic morphological changes and are believed to support breastmilk production. Gap junctions, predominantly consisting of Cx43 protein in the mammary adipose tissue, may allow signal transduction between adipocytes or between adipocytes and other types of cells. Previous publications have demonstrated that adipocyte Cx43 is reduced in male mice with obesity and is implicated in the development of metabolic dysfunction [12]. Therefore, in this study, we sought to understand whether adipocyte Cx43 regulates mammary tissue remodeling to affect breastmilk quality.

We first confirmed a reduction in adipocyte Cx43 gap junction mRNA and protein levels in the female mice fed with HFD. Given the strong inverse correlation between Cx43 expression and adipocyte lipid accumulation [26,27], a phenomenon also observed in skeletal muscle cells [28], osteoblasts [29], bone marrow cells [30], and the heart [31], we hypothesized that a reduction in adipocyte Cx43 expression in female mice would make adipocytes resistant to delipidation and compromise the adipose tissue’s remodeling to support breastmilk production. We used a doxycycline-inducible adipocyte-specific Cx43 knockout (Apn-Cx43-KO) mouse model and found that the basal metabolic phenotype of Apn-Cx43-KO mice on a chow diet was the same as that of control mice, reflecting Cx43’s dispensable role in the metabolic regulation of mature adipocytes. We postulate that the adipocyte Cx43 gap junction and active intercellular communications may only be needed during dynamic adipocyte remodeling. Indeed, when mice were undergoing pregnancy and lactation, the deletion of Cx43 in the adipocyte impaired lipid mobilization of adipocytes, and breastmilk extracted from Apn-Cx43-KO mice showed lower lactose concentration. Together with the lack of systemic metabolic defects, these data suggest that adipocyte Cx43 per se plays a vital role in breastmilk macronutrient production. We also observed a lower body weight of cross-fostered pups nourished by the Apn-Cx43-KO dams, suggesting that their breastmilk is less efficient in supporting pups’ growth. While there is a report showing that human milk lactose concentration is lower in obese women four months postpartum [32], many other studies fail to observe a difference in lactose concentrations [33], highlighting considerable variability in the results between human studies. Whether the slower neonates’ growth was solely caused by reduced lactose concentration in the breastmilk remains to be investigated. Future endeavors will examine whether the adipocyte-specific overexpression of Cx43 will improve adipocyte remodeling and breastmilk quality in supporting neonates’ growth.

There have been many epidemiological studies on childhood famine and late-life metabolic syndrome [34], highlighting the importance of improving neonatal nutrition to support childhood development and health in adulthood [35]. While pharmaceutical companies are unlikely to develop a drug to increase adipocyte Cx43 expression in obese women to improve their breastmilk production, due to very high safety requirements for such a drug, and the relatively good performance and safety profile of baby formula on the market, some nutritional supplements, such as Genistein and Quercetin [36], may be used to increase Cx43 expression. For example, Genistein has been shown to increase Cx43 expression in the prefrontal cortex of depressed mice, improving their symptoms [37]. It is, therefore, worth testing in the future whether Genistein can also increase adipocyte Cx43 expression and whether it will enhance the quality of breastmilk in lactating obese women. Capsaicin, the active component of chili peppers, is another compound proven to increase adipocyte Cx43 expression [38]. For example, a cell culture and rodent study provided direct evidence that capsaicin treatment increases Cx43 protein in 3T3-L1 preadipocytes and improves the metabolic function of mice with diet-induced obesity [38]. While ingesting chili peppers may affect the flavor of breastmilk, research indicates that lactating mothers need not avoid any specific food unless the infant reacts negatively to it [39]. Therefore, one future endeavor will be to test whether spicy food intake helps improve mothers’ metabolic function during pregnancy and breastmilk quality to support their neonates.

## 5. Conclusions

Adipocyte Cx43 gap junctions are critical cellular structures facilitating adipocyte remodeling in breastmilk production. Endeavors to enhance Cx43 expression in adipocytes would help lactating women produce better breastmilk.

## Figures and Tables

**Table 1:** List of primers used in the paper.

Name of the Gene	PCR Product Length	Name of the Primer	Sequence (5′ to 3′)
*Gja1*	158 bp	forward	GTCACTGGTGACAGAAACAA
		reverse	CTGTCGTCAGGGAAATCAAA
*Gja4*	220 bp	forward	CCCACATCCGATACTGGGTG
		reverse	CGAAGACGACCGTCCTCTG
*Gjb1*	134 bp	forward	GCACGTAGCTCACCAACAG
		reverse	TGATGACATAGGTCCACCACA
*Gjb2*	133 bp	forward	ATCCTCGGGGGTGTCAACAA
		reverse	AGACAAAATCGGCTTGCTCATC
*Gjc1*	167 bp	forward	CAGAGCCAACCAAAACCTAAGC
		reverse	CTGCACACATAAAATGGGTGGA
*Rps16*	127 bp	forward	CACTGCAAACGGGGAAATGG
		reverse	CACCAGCAAATCGCTCCTTG
*Rps18*	174 bp	forward	GGAGATATGCTCATGTGGTG
		reverse	TGTACTTCCCATCCTTCACA

## Data Availability

All data supporting the findings of this study are available upon reasonable request. Mouse models are available from the corresponding author upon request.

## Supplementary Materials

The following are available online at https://www.mdpi.com/article/10.3390/biology11071023/s1, Figure S1: The original blot of Figure 2C. The Odyssey® One-Color Protein Molecular Weight Marker (Li-Cor, P/N: 928-40000, Lincoln, NE, USA) is shown in red, the molecular weights are in kilodaltons. Only *Gja1*^fl/fl^ groups on HFD and Chow (yellow rectangle) were presented in the Figure 2C. Densitometry of Cx43 (green channel) normalized by Actin (red channel) is shown below each band. Figure S2: The original blot of Figure 3B. The Odyssey® One-Color Protein Molecular Weight Marker (Li-Cor, P/N: 928-40000, Lincoln, NE, USA) is shown in red, the molecular weights are in kilodaltons. Densitometry of Cx43 (red channel) normalized by Tubulin (green channel) is shown below each band.
