# Supplementary material for: Maternal Adipocyte Connexin43 Gap Junctions Affect Breastmilk Lactose Levels and Neonate Growth in Mice
Source: Biology (Basel). 2022 Jul 7;11(7):1023. doi: 10.3390/biology11071023 (PMC9311998; doi:10.3390/biology11071023)
Supplement: Supplementary file 1 [file biology-11-01023-s001.zip › biology-1786328-supplementary.pdf]

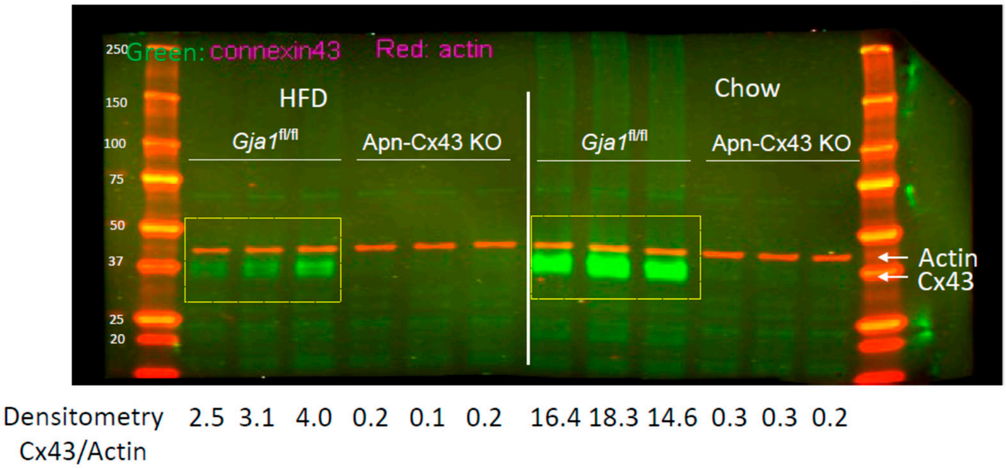

Figure S1. The original blot of Fig. 2C. The Odyssey® One-Color Protein Molecular Weight Marker (Li-Cor, P/N: 928-40000, Lincoln, NE) is shown in red, the molecular weights are in kilodaltons. Only *Gja1*$^{fl/fl}$ groups on HFD and Chow (yellow rectangle) were presented in the Fig. 2C. Densitometry of Cx43 (green channel) normalized by Actin (red channel) is shown below each band.

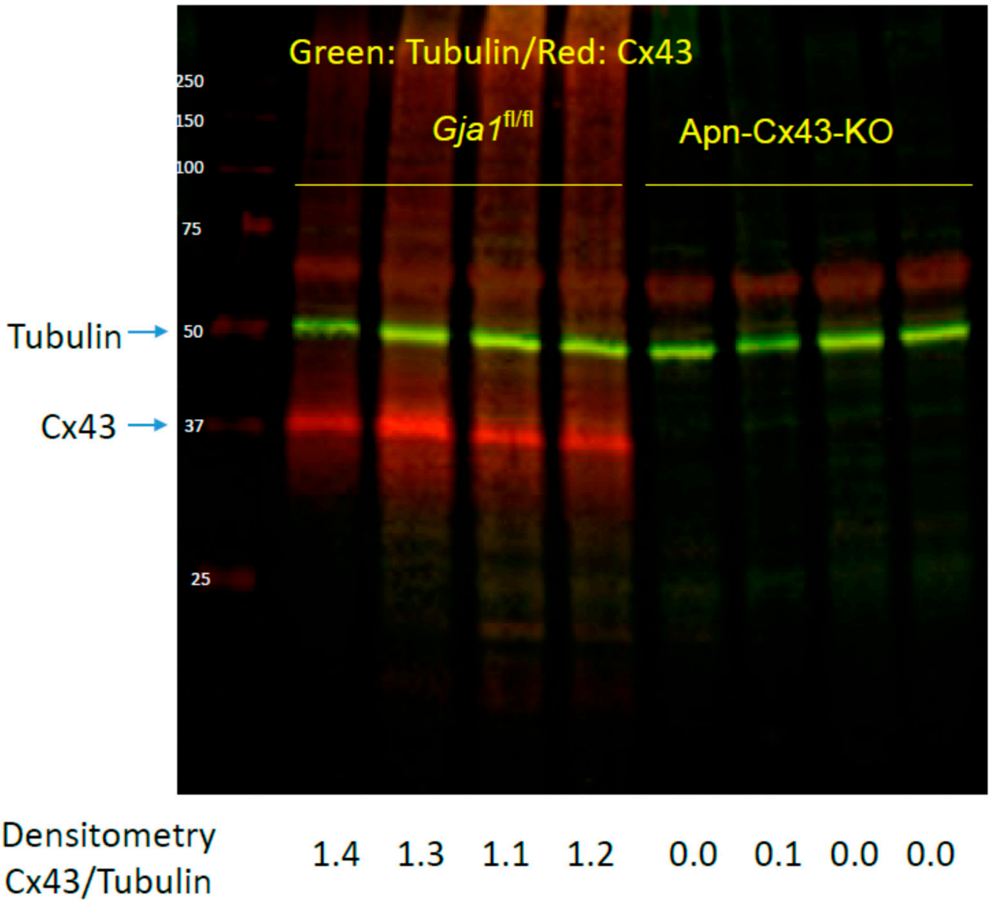

Figure S2. The original blot of Fig. 3B.The Odyssey® One-Color Protein Molecular Weight Marker (Li-Cor, P/N: 928-40000, Lincoln, NE) is shown in red, the molecular weights are in kilodaltons. Densitometry of Cx43 (red channel) normalized by Tubulin (green channel) is shown below each band.
